# Abundance and Diversity of Denitrifying and Anammox Bacteria in Seasonally Hypoxic and Sulfidic Sediments of the Saline Lake Grevelingen

**DOI:** 10.3389/fmicb.2016.01661

**Published:** 2016-10-20

**Authors:** Yvonne A. Lipsewers, Ellen C. Hopmans, Filip J. R. Meysman, Jaap S. Sinninghe Damsté, Laura Villanueva

**Affiliations:** ^1^Department of Marine Microbiology and Biogeochemistry, Royal Netherlands Institute for Sea Research, Utrecht UniversityDen Burg, Netherlands; ^2^Department of Estuarine and Delta Systems, Royal Netherlands Institute for Sea Research, Utrecht UniversityDen Burg, Netherlands; ^3^Faculty of Geosciences, Department of Earth Sciences, Utrecht UniversityUtrecht, Netherlands

**Keywords:** anammox bacteria, denitrifiers, sulfide-oxidizing bacteria, *nirS* gene, *aprA* gene, intact polar lipids (IPL), ladderane lipid

## Abstract

Denitrifying and anammox bacteria are involved in the nitrogen cycling in marine sediments but the environmental factors that regulate the relative importance of these processes are not well constrained. Here, we evaluated the abundance, diversity, and potential activity of denitrifying, anammox, and sulfide-dependent denitrifying bacteria in the sediments of the seasonally hypoxic saline Lake Grevelingen, known to harbor an active microbial community involved in sulfur oxidation pathways. Depth distributions of 16S rRNA gene, *nirS* gene of denitrifying and anammox bacteria, *aprA* gene of sulfur-oxidizing and sulfate-reducing bacteria, and ladderane lipids of anammox bacteria were studied in sediments impacted by seasonally hypoxic bottom waters. Samples were collected down to 5 cm depth (1 cm resolution) at three different locations before (March) and during summer hypoxia (August). The abundance of denitrifying bacteria did not vary despite of differences in oxygen and sulfide availability in the sediments, whereas anammox bacteria were more abundant in the summer hypoxia but in those sediments with lower sulfide concentrations. The potential activity of denitrifying and anammox bacteria as well as of sulfur-oxidizing, including sulfide-dependent denitrifiers and sulfate-reducing bacteria, was potentially inhibited by the competition for nitrate and nitrite with cable and/or *Beggiatoa*-like bacteria in March and by the accumulation of sulfide in the summer hypoxia. The simultaneous presence and activity of organoheterotrophic denitrifying bacteria, sulfide-dependent denitrifiers, and anammox bacteria suggests a tight network of bacteria coupling carbon-, nitrogen-, and sulfur cycling in Lake Grevelingen sediments.

## Introduction

Nitrogen availability is a major factor controlling primary production in temperate coastal marine environments with high anthropogenic nitrogen input (Herbert, 1999). Denitrification is a key process in the nitrogen cycle of coastal sediments releasing gaseous end products, nitric oxide (NO), nitrous oxide (N_2_O), and dinitrogen gas (N_2_) to the atmosphere. This nitrogen removal can result in a decrease of nitrogen availability for primary producers and thereby controlling the rate of eutrophication in coastal marine systems (Seitzinger, 1998; Herbert, 1999). Multiple microbial mediated pathways result in the removal of nitrogen in anoxic sediments (see Devol, 2015 for a detailed review). Here we focus on (1) denitrification, the stepwise conversion of nitrate/nitrite to dinitrogen gas which is mainly performed by facultative organoheterotrophic anaerobic bacteria and some archaea (Zumft, 1997); (2) anaerobic ammonium oxidation (anammox), the oxidation of ammonium with nitrite to dinitrogen gas carried out by anammox bacteria (Kuypers et al., 2003); and (3) sulfide-dependent denitrification, the oxidation of sulfide with nitrate performed by autotrophic members of α-, β-, γ-, and ε-proteobacteria, which could contribute to denitrification and to the removal of sulfide in the oxygen transition zone of coastal marine sediments (Shao et al., 2010).

Numerous environmental factors, such as the availability of nitrogen speciation and concentration, temperature, oxygen concentrations, organic matter quality and quantity, bioturbation, and other sediment characteristics have been suggested to affect the distribution and abundance of denitrifying and anammox bacteria (Thamdrup and Dalsgaard, 2002; Meyer et al., 2005; Jensen et al., 2008; Dang et al., 2010; Laverock et al., 2013; Prokopenko et al., 2013; Babbin et al., 2014; Zhang et al., 2014). In this study, we assessed the effects of hypoxia and elevated sulfide concentration on the abundance and activity of denitrifiers and anammox bacteria in marine sediments as these factors have been previously suggested as potential limiting factors in denitrification processes (Brunet and Garcia-Gil, 1996; Burgin and Hamilton, 2007; Aelion and Warttinger, 2010; Neubacher et al., 2011, 2013; Bowles et al., 2012). Seasonal hypoxia is an increasing phenomenon that occurs in coastal areas causing a decrease in the electron acceptors (O_2_, ${\text{NO}}_{3}^{-}$) in the bottom waters (Diaz and Rosenberg, 2008). Besides, sulfide inhibition can decrease denitrification rates as the enzyme catalyzing the reduction of nitrous oxide to N_2_ is sensitive to sulfide (e.g., Porubsky et al., 2009). In addition, several studies have provided putative evidence indicating that sulfide inhibits the anammox reaction. For example, Dalsgaard et al. (2003) reported a decrease in anammox activity in the sulfidic waters of the anoxic basin of Golfo Dulce (Costa Rica). Also Jensen et al. (2008) showed that sulfide had a direct inhibiting effect on the activity of anammox bacteria in the Black Sea.

In this study, we evaluated the impact of environmental factors, such as oxygen and free sulfide concentrations in the diversity, abundance, activity, and spatial distribution of bacteria involved in N_2_ removal pathways in sediments of Lake Grevelingen (The Netherlands), a seasonally hypoxic saline reservoir. Here, we determined the diversity, abundance and potential activity of denitrifying bacteria, anammox bacteria and sulfur-oxidizing bacteria (SOB), including sulfide-dependent denitrifiers, and sulfate-reducing (SRB), in three different stations within the lake both in March (before hypoxia) and August (i.e., during hypoxia). In order to determine changes in the diversity, abundance and activity of denitrifiers, we targeted the *nirS* gene, encoding for the cytochrome *cd*_1_ nitrite reductase, catalyzing nitrite reduction to nitric oxide (NO; Braker and Fesefeldt, 1998; Smith et al., 2007; Huang et al., 2011). Here, we focused on the cytochrome *cd*1-containing nitrite reductase (*nirS* gene), as it has been found to be more widespread in the bacterial communities compared to the copper-containing nitrite reductase (*nirK*) in various sediments (Braker et al., 1998; Priemé et al., 2002; Liu et al., 2003; Throbäck et al., 2004; Tiquia et al., 2006; Oakley et al., 2007; Dang et al., 2009; Huang et al., 2011). For anammox bacteria, the *nir*S gene was also quantified as it has been recently suggested to be a functional biomarker anammox bacteria (Li et al., 2011). Both, denitrifying and anammox bacteria harbor one copy of the *nirS* gene, indicating that the *nirS* gene might be a suitable marker to compare the abundance and distribution of *nirS*-type denitrifiers and anammox bacteria in coastal sediments. The potential activity of denitrifying and anammox bacteria was also determined by estimating the gene expression of those metabolic genes as in previous studies (Smith et al., 2007; Lam et al., 2009; Bale et al., 2014; Bowen et al., 2014; Lipsewers et al., 2014; Zhang et al., 2014). Besides functional genes, the abundance of anammox bacteria was also determined by the quantification of ladderane lipids, which are specific lipid biomarkers for this microbial group (Sinninghe Damsté et al., 2002; Jaeschke et al., 2009; Russ et al., 2013). Finally, the diversity and abundance of sulfur-oxidizing and sulfate-reducing bacteria in marine sediments was estimated by targeting the *aprA* gene encoding the adenosine-5′-phosphosulfate (APS) reductase (Blazejak and Schippers, 2011; Lenk et al., 2011; Dyksma et al., 2016). The APS reductase is operating in in SOB, oxidizing sulfite to APS, as well as in SRB, operating in reverse direction, converting APS to adenosine monophosphate (AMP) and sulfite (${\text{SO}}_{3}^{2-}$) (Meyer and Kuever, 2007b).

Our starting hypothesis is that the abundance and potential activity of organoheterotrophic denitrifiers and anammox bacteria would decrease upon increase of the sulfide concentration found in the sediments during the summer hypoxia. Moreover, recent studies point out that sulfide-dependent denitrifiers play a relevant role in the sulfide transition zone of intertidal sediments (Dyksma et al., 2016). Therefore, we hypothesize that their abundance and activity would be higher in hypoxic and sulfidic conditions, thus contributing more to the general N_2_ removal in comparison to organoheterotrophic denitrifying and anammox bacteria and eventually being involved in sulfide detoxification which could promote anammox activity.

## Materials and methods

### Study site and sampling

Lake Grevelingen is a former estuary within the Scheld-Rhine-Meuse delta, and was formed by the construction of the Grevelingendam on the landside in 1964 and the Brouwersdam on the seaward side in 1971. The lake (surface area: 108 km^2^, mean water depth 5.3 m) mainly consists of shallow water areas with the exception of the former tidal gullies, that have a water depth of up to 48 m (Keldermann et al., 1984; Nienhuis and De Bree, 1984). After its closure, the lake transformed into a freshwater body, but in 1979, the connection with the North Sea was partially re-established, and since then, Lake Grevelingen has a high and relatively constant salinity (29−32). Within Lake Grevelingen, the Den Osse basin forms a deeper basin within the main gully, and experiences a regular seasonal stratification leading to oxygen depletion in the bottom water (summer hypoxia; Wetstejn, 2011). Due to sediment focusing, the Den Osse basin (maximum water depth 34 m) also experiences a rapid accumulation of fine-grained, organic rich sediments (sediment accumulation rate ~2 cm yr^−1^; Donders and Guasti, 2011).

Sediment cores were collected along a depth gradient in Den Osse basin during two cruises March and August 2012 on board of the *RV* Luctor. Sampling took place at three different stations: S1 was located in the deepest point of the basin at 34 m water depth (51.747°N, 3.890°E), S2 at 23 m (51.749°N, 3.897°E) and S3 at 17 m (51.747°N, 3.898°E). Sediment was collected with single core gravity corer (UWITEC) using transparent PVC core liners (6 cm inner diameter, 60 cm length). Four sediment cores were collected at each station in March and in August. The cores were sliced with a 1 cm resolution until 5 cm depth, and sediment samples were collected for lipid and DNA/RNA analysis and kept at −80°C until further processing. In each sampling campaign, a water column depth profile of temperature, salinity and oxygen (O_2_) concentration was recorded at S1 using an YSI 6600 CTD instrument (for details see Hagens et al., 2014). Oxygen concentrations recorded by the CTD instrument were calibrated based on discrete water samples using an automated Winkler titration procedure (Knap et al., 1996). Bottom water concentrations of ammonium (${\text{NH}}_{4}^{+}$), nitrite (${\text{NO}}_{2}^{-}$) and nitrate (${\text{NO}}_{3}^{-}$) were measured colometrically on a SEAL QuAAtro segmented flow nutrient analyzer. Monitoring data at Lake Grevelingen (Wetstejn, 2011) shows that the water column is laterally homogenous over the Den Osse basin scale, which allows estimation of the bottom water parameters at S2 and S3 from corresponding depths in the measured CTD profiles at station S1.

### Sediment geochemistry

High-resolution depth profiles of O_2_ and sulfide (H_2_S) were measured in intact sediment cores to determine the oxygen penetration depth (OPD) and the sulfide appearance depth (SAD), using commercial micro-electrodes (Unisense A.S., Denmark) operated with a motorized micromanipulator (for details on the procedure see Malkin et al., 2014). The OPD is operationally defined as the depth below which [O_2_] <1 μM, while the sulfide appearance depth (SAD) is operationally defined as the depth below which [H_2_S] >1 μM (Seitaj et al., 2015).

Sediment cores were sectioned in increments of 0.5 cm from the sediment-water interface to 5 cm depth, and pore water was extracted by centrifugation, and analyzed following the procedure of (Sulu-Gambari et al., 2016). After filtration through 0.22 μm cellulose filters (Chromafil Xtra), pore water samples were analyzed for total free sulfide (∑H_2_S) (Cline, 1969; standard deviation ± 0.4 μM), whereas ammonium (${\text{NH}}_{4}^{+}$)via spectrophotometry was determined by a SEAL QuAAtro segmented flow analyzer (Aminot et al., 2009) after a 25 times dilution with a low nutrient seawater matrix solution (standard deviation ± 3.5%).

The free sulfide and ammonium concentration depth profiles of the sediment pore water were averaged to provide a 1 cm resolution down to 5 cm sediment depth to enable a direct comparison with results of the DNA/RNA and lipid analysis. The total organic carbon (TOC) content of the sediment was determined on sediment samples that were freeze-dried, ground to a fine powder and analyzed by an a Thermo Finnigan Delta plus isotope ratio monitoring mass spectrometer (irmMS) connected to a Flash 2000 elemental analyzer (Thermo Fisher Scientific, Milan). Before the analysis, samples were first acidified with 2N hydrogen chloride (HCl) to remove the inorganic carbon (Nieuwenhuize et al., 1994). Concentrations of TOC are expressed as mass % of dry sediment.

### DNA/RNA extraction

DNA and RNA from sediments (previously centrifuged to remove excess of water thus values are given as grams of wet weight; S1, S2, and S3; 0–5 cm sediment depth; 1 cm resolution) were extracted by using the DNA and RNA PowerSoil® Total Isolation Kit, respectively (Mo Bio Laboratories, Inc., Carlsbad, CA). Nucleic acid concentrations were quantified spectrophotometrically (Nanodrop, Thermo Scientific, Wilmington, DE) and checked by agarose gel electrophoresis for integrity. Extracts were kept frozen at −80°C. The RNA extracts were treated with RNase-free DNase (DNA-*free*™, Ambion Inc., Austin, TX), and RNA quality and concentration were estimated by the Experion RNA StdSens Analysis Kit (Bio-Rad Laboratories, Hercules, CA). DNA contamination was checked by PCR using RNA as a template. Reverse transcription was performed as specified in Lipsewers et al. (2014).

### PCR amplification and cloning

Amplifications of the *nirS* gene of denitrifying bacteria (S1, S2, S3, March and August, 0–1 cm), and anammox bacteria 16S rRNA gene (S2, March, 1–2 cm), specific *nirS* genes of *Scalindua* sp. (S3, August, 0–1 cm) and the *aprA* gene (S2, August, 0–1 cm) were performed with the primer pairs specified in Table S1. The PCR reaction mixture consisted of (final concentration): Q-solution (PCR additive, Qiagen, Valencia, CA) 1 × ; PCR buffer 1 × ; BSA (200 μg ml^−1^); dNTPs (20 μM); primers (0.2 pmol μl^−1^); MgCl_2_ (1.5 mM); 1.25 U Taq polymerase (Qiagen, Valencia, CA). PCR conditions for these amplifications were: 95°C, 5 min; 35 × [95°C, 1 min; Tm, 1 min; 72°C, 1 min]; final extension 72°C, 5 min. PCR products were gel purified (QIAquick gel purification kit, Qiagen, Valencia, CA) and cloned in the TOPO-TA cloning® kit (Life Technologies, Carlsbad, CA) and transformed in *E. coli* TOP10 cells following the manufacturer's recommendations. In addition, in order to test the specificity of the quantitative PCR reaction we repeated the reactions of anammox bacteria 16S rRNA gene (Broc541F-Amx820R) with DNA extract of S2, March, 0–1 cm, *nirS* gene of heterotrophic denitrifying bacteria (nirS1F-nirS3R) with cDNA of S2, March, 0–1 cm, and *aprA* gene (Apr1F- Apr5R) with cDNA of S2, August, 0–1 cm, which were then treated to add 3′-A-overhangs and then cloned with the TOPO-TA cloning® kit as indicated above. Recombinant plasmid DNA was sequenced using the M13R primer by Macrogen Inc. (Amsterdam, The Netherlands).

### Phylogenetic analysis

Sequences were analyzed for the presence of chimeras using the Bellerophon tool at the GreenGenes website (http://greengenes.lbl.gov/). Sequences were aligned with MEGA6 software (Tamura et al., 2013) by using the alignment method ClustalW. The phylogenetic trees of the *nirS* and *aprA* genes were computed with the Neighbor-Joining method (Saitou and Nei, 1987) using the Poisson model with a bootstrap test of 1000 replicates. The phylogenetic affiliation of the partial anammox bacteria 16S rRNA gene sequences was compared to release 123 of the SILVA NR SSU Ref database (http://www.arb-silva.de/; Quast et al., 2013) using the ARB software package (Ludwig et al., 2004). Sequences were added to the reference tree supplied by the SILVA database using the ARB Parsimony tool. Sequences were deposited in NCBI with the following accession numbers: KP886533–KP886678 for *nirS* gene sequences of denitrifiers, KP886679–KP8866700 for 16S rRNA gene sequences of anammox bacteria, KP886701–KP886721 for *nirS* gene sequences of anammox bacteria and KP886722–KP886804 for *aprA* gene sequences of SOB and SRB.

### Quantitative PCR (qPCR) analysis

qPCR analyses were performed on a Biorad CFX96™ Real-Time System/C1000 Thermal cycler equipped with the CFX Manager™ software for sediment DNA/RNA extracts (S1, S2, and S3; 0–5 cm sediment depth; 1 cm resolution). Detailed information about the primers used in this study are summarized in Table S1. The abundance of denitrifying bacteria specific *nirS* gene was quantified using the primer set nirS1F/nirS3R as described by Braker and Fesefeldt (1998). The abundance of anammox bacteria 16S rRNA gene was estimated using primers Brod541F/Amx820R as described by Li et al. (2010). Additionally, a fragment of the *Scalindua* sp. specific *nirS* gene, which codes for the cytochrome *cd*1-containing nitrite reductase, was quantified using the primer combination Scnir372F/Scnir845R as described by Lam et al. (2009). The abundance of SOB and SRB including sulfide dependent denitrifiers was estimated by targeting the dissimilatory adenosine-5′-phosphosulfate (APS) reductase (*aprA* gene) involved in the APS reduction of SOB and in the sulfite oxidation of sulfate-reducing bacteria by using the primer combination Apr-1-FW/Apr-5-RW as described by Meyer and Kuever (2007a) (see Table S1 for details). Gene abundances are expressed as copies g^−1^ sediment of wet weight.

All qPCR amplifications were performed in triplicate with standard curves ranging from 10^0^ to 10^7^ molecules per microliter. Standard curves and qPCR amplifications were performed as previously described by Lipsewers et al. (2014). Coefficients of determination (*R*^2^) for standard curves ≥0.998 and qPCR efficiencies (E) ≥80% were accepted.

### Anammox bacteria ladderane lipid analysis

Intact polar lipids (IPLs) were extracted with the Bligh and Dyer extraction method (Bligh and Dyer, 1959; mod. by Pitcher et al., 2011) as described in detail by Bale et al. (2014). Intact ladderane phospholipids specific for anammox bacteria, the C_20_-[3]-monoether ladderane attached to a phosphatidylcholine (PC) headgroup (PC-monoether ladderane) was analyzed by HPLC-MS/MS following Jaeschke et al. (2009) and quantified using an external standard consisting of isolated PC-monoether ladderane. Sediment samples between 0 and 5 cm depth (1 cm resolution) were analyzed and PC-monoether ladderane lipid concentrations were expressed per nanogram of dry weight sediment (ng g^−1^). In order to determine the fatty acid composition of the ladderane lipids, aliquots of the Bligh and Dyer extracts (BDE) obtained from the 0 to 1 and 4 to 5 cm sediment layers were saponified by reflux with aqueous KOH (in 96% MeOH) for 1 h. Fatty acids were obtained by acidifying the saponified samples to a pH of 3 with 1N HCl in MeOH and extracted using dichloromethane (DCM). The fatty acids were converted to their corresponding fatty acid methyl esters (FAMEs) by methylation with diazomethane (CH_2_N_2_) as described by Rush et al. (2012a). Polyunsaturated fatty acids (PUFAs) were removed by eluting the sample over a silver nitrate (AgNO_3_) (5%) impregnated silica column with DCM and air-dried at room temperature. The fatty acid fractions were dissolved in acetone, filtered through a 0.45 μm polytetrafluoroethylene (PTFE) filters (4 mm diameter), and analyzed by high performance liquid chromatography coupled to positive ion atmospheric pressure chemical ionization tandem mass spectrometry (HPLC/APCI-MS/MS) in selective reaction monitoring (SRM) mode following Hopmans et al. (2006) including the recent modifications described by Rush et al. (2012b). Ladderane lipids were quantified using external calibration curves of three standards of isolated methylated ladderane fatty acids (C20-[3]-ladderane fatty acid, and C20-[5]-ladderane fatty acid; Hopmans et al., 2006; Rush et al., 2011).

## Results

Sediment samples were collected along a depth gradient in Den Osse basin (Lake Grevelingen) during two cruises March (before summer hypoxia) and August (during summer hypoxia) 2012. Sampling took place at three different stations: S1 was located in the deepest point of the basin at 34 m water depth, S2 at 23 m and S3 at 17 m.

### Environmental conditions

The temperature of the bottom water at station S1 (Figure S1) showed a regular seasonal cycle with lowest values in late winter (1.5°C in February) and highest values in late summer (16.9°C in September). During the spring campaign (March 2012), the water column was only partially stratified and showed a limited surface-to-bottom temperature gradient. In contrast, during the summer campaign (August 2012), the water column was thermally stratified. The yearly pattern of the bottom water oxygenation at station S1 was inversely correlated the temperature, with greatest oxygenation levels in winter and fall, and lowest concentrations in summer (Figure S1). In March 2012, the bottom water oxygenation was similar for all three stations, whereas in August 2012, the bottom water at S1 and S2 were anoxic (<1 μM), while the bottom water at S3 still had 88 μM of O_2_ (36% air saturation). Bottom water ammonium (${\text{NH}}_{4}^{+}$) concentrations in station S1 ranged from 3 μM in March to 11.5 μM in August; nitrite (NO${\text{NO}}_{2}^{-}$) concentrations were relatively constant (0.7–1 μM) in March and August. Nitrate concentrations ranged from 28 μM in March to <2 μM in August in station S1 (Figure S1) whereas in stations S2 and S3 values varied between 28 μM in March to ~10 μM in August.

The OPD in the sediment was seasonally variable and increased from S1 to S3, i.e., in S1 in March OPD was 1.5 mm and in August the sediment was completely anoxic. In S2, OPD was between 1.7 and 2.5 mm in March and in August ca. 0.5 mm (hypoxic) and in S3, the OPD was between 1.5 and 2.2 mm in March and ca. 1.0 mm (hypoxic) in August. (Table 1). The sulfide appearance depth (SAD) varied between March and August in all stations. The SAD moved toward the sediment surface between March and August in all stations. In March, the SAD in stations S1 and S2 was at 18.4 and 21.3 mm, respectively, whereas in S3, the SAD was detected at 41.8 mm sediment depth. However, in August, SAD in stations S1 and S2 was at 0.4 and 0.6 mm, respectively. In station S3, the sulfide was detected at 4.2 mm sediment depth (Table 1). At station S2, white mats of *Beggiatoa* sp.-like microorganisms covered the sediment surface in March. Small polychaetes were observed in the sediment at station S3 in March, suggesting some bioturbation. Sulfide (∑H_2_S) concentrations in the sediment pore water were low in March in all three stations, i.e., ranging from 0 to 0.007 mM, whereas in comparison, all three stations showed high sulfide concentrations in August, i.e., 0.15–1.6 mM (Table 1; see Seitaj et al., 2015 for detailed geochemical profiles of station S1). Ammonium concentrations were low in March, i.e., ranging from 0.24 to 0.63 mM on average, in comparison with August when ${\text{NH}}_{4}^{+}$ concentrations reached higher values, i.e., 0.7–1.2 mM on average (Table 1). The TOC content of the sediments varied slightly between stations and seasons, ranging between 1.8 and 4.4% (Table S2).

**Table 1:** **Sediment porewater ammonia (${\text{NH}}_{4}^{+}$) and sulfide (HS^**−**^) concentrations (1 cm resolution), oxygen penetration depth (OPD), and sulfide appearance depth (SAD) determined by micro-sensor profiling**.

**Station**	**Sediment depth (cm)**	HS^−^(μM)^*^		${\text{NH}}_{4}^{+}$ (μM)^*^		OPD (mm)^**^		SAD (mm)^**^	
		**March**	**August**	**March**	**August**	**March**	**August**	**March**	**August**
1	0–1	0	810	279	656	1.5	0	18.4	0.4
	1–2	0	1503	410	1071				
	2–3	0	1639	636	1322				
	3–4	0	1962	833	1567				
	4–5	33	2063	979	1768				
2	0–1	0	1157	165	550	1.7	2.5	21.3	0.6
	1–2	0	802	455	836				
	2–3	0	1008	526	1027				
	3–4	2	1238	592	1138				
	4–5	27	1190	671	1228				
3	0–1	0	211	73	537	1.5	2.2	41.8	4.2
	1–2	0	177	154	694				
	2–3	0	146	236	736				
	3–4	0	109	333	749				
	4–5	0	93	408	746				

* *Data are averaged to reach a 1 cm resolution; data provided by Sulu-Gambari et al. (2016; unpublished data)*,

** *data provided by Seitaj et al. (2015; unpublished data)*.

### Diversity, abundance, and potential activity of *nirS*-type denitrifiers

**Figure 1:**
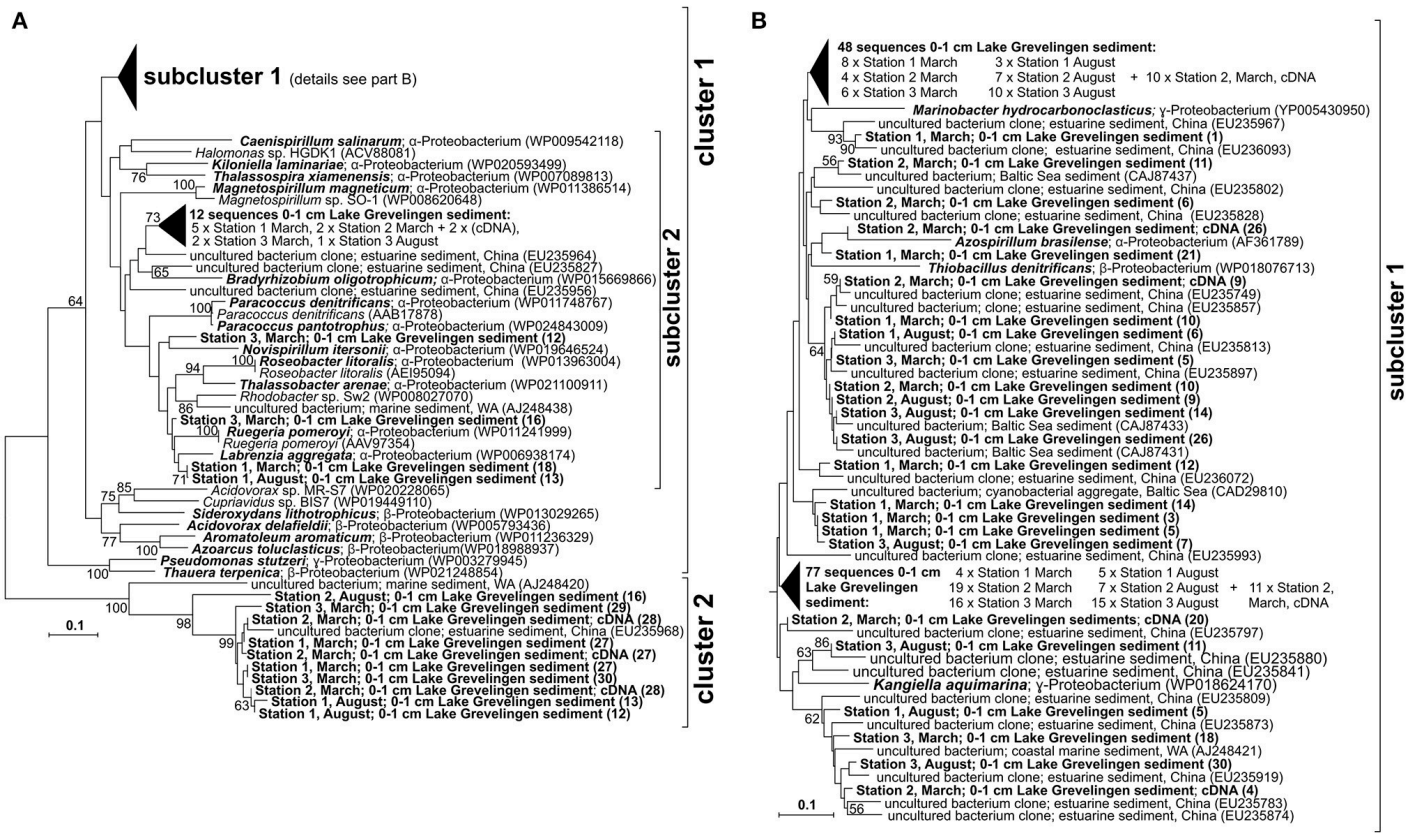
**(A)** Phylogenetic tree of partial *nirS* gene sequences of denitrifying bacteria retrieved in this study, **(B)** detail of subcluster 1 as indicated in **(A)**. 147 DNA sequences recovered from stations S1, S2, and S3 between 0 and 1 cm sediment depth, in March and August by amplification (PCR) and cloning and 28 cDNA sequences obtained from station S2 between 0 and 1 cm sediment depth in March recovered by amplification (qPCR) and cloning) and closest relatives (bold: our sequences and closest known relatives); the scale bar indicates 25% sequence divergence.

In our study, we focused on heterotrophic and autotrophic bacteria that are able to perform the dissimilatory reduction of nitrite to nitric oxide. This reaction forms an intermediate step in the complete denitrification of nitrate to N_2_ and is catalyzed by the cytochrome *cd*1-containing nitrite reductase encoded by the *nirS* gene (Braker et al., 2000). The diversity of *nirS*-type denitrifiers was evaluated for the surface sediment layer (0–1 cm) at the three stations in March and August by phylogenetic analysis targeting the *nirS* gene (Figures 1 A,B). In general, the *nirS* sequences obtained (147 sequences in total) were closely related to *nirS* sequences of uncultured organisms found in coastal marine environments with a high input of organic matter such as estuarine sediments and eutrophic bay sediments (Braker et al., 2000, Zhang et al., 2014). The phylogenetic analysis of protein sequences of the *nirS* gene revealed two distinct clusters (Figure 1A; cluster 1 and 2), where most (ca. 95%) of the sequences clustered in cluster 1 (ca. 95%). Within cluster 1, ~90% of the *nirS* gene sequences were grouped into subcluster 1.1 and 10% into subcluster 1.2 (Figures 1 A,B; Figure S2). Within subcluster 1.1 sequences were affiliated to *nirS* sequences of members of α-, β-, and γ-proteobacteria able to perform autotrophic denitrification coupled to sulfide oxidation (*Thiobacillus denitrificans*) and heterotrophic denitrification (*Azospirillum brasilense, Marinobacter hydrocarbonoclasticus, Kangiella aquamirina, Halomonas* sp.). Sequences grouped in subcluster 1.2 were affiliated to the *nirS* gene sequences of divers α- proteobacteria able to perform autotrophic denitrification coupled to sulfide or iron oxidation (*Paracoccus denitrificans, Sideroxidans lithotrophicus*), as well as heterotrophic denitrification (e.g., *Aromatoleum aromaticum, Azocarus toluclasticus, Acidovorax delafieldii*). Sequences grouped into cluster 2 were affiliated to *nirS* gene sequences of uncultured bacteria detected in coastal marine and estuarine sediments (Braker et al., 2000; Zhang et al., 2014). In order to determine the specificity of the qPCR assay, sequences of *nirS* cDNA (complementary DNA of *nirS* mRNA) generated during the qPCR reaction were cloned and sequenced (28 sequences in total) and also added to the protein-coding *nirS* sequences phylogenetic tree showing that those sequences were grouped in the clusters described before (Figures 1 A,B).

**Figure 2:**
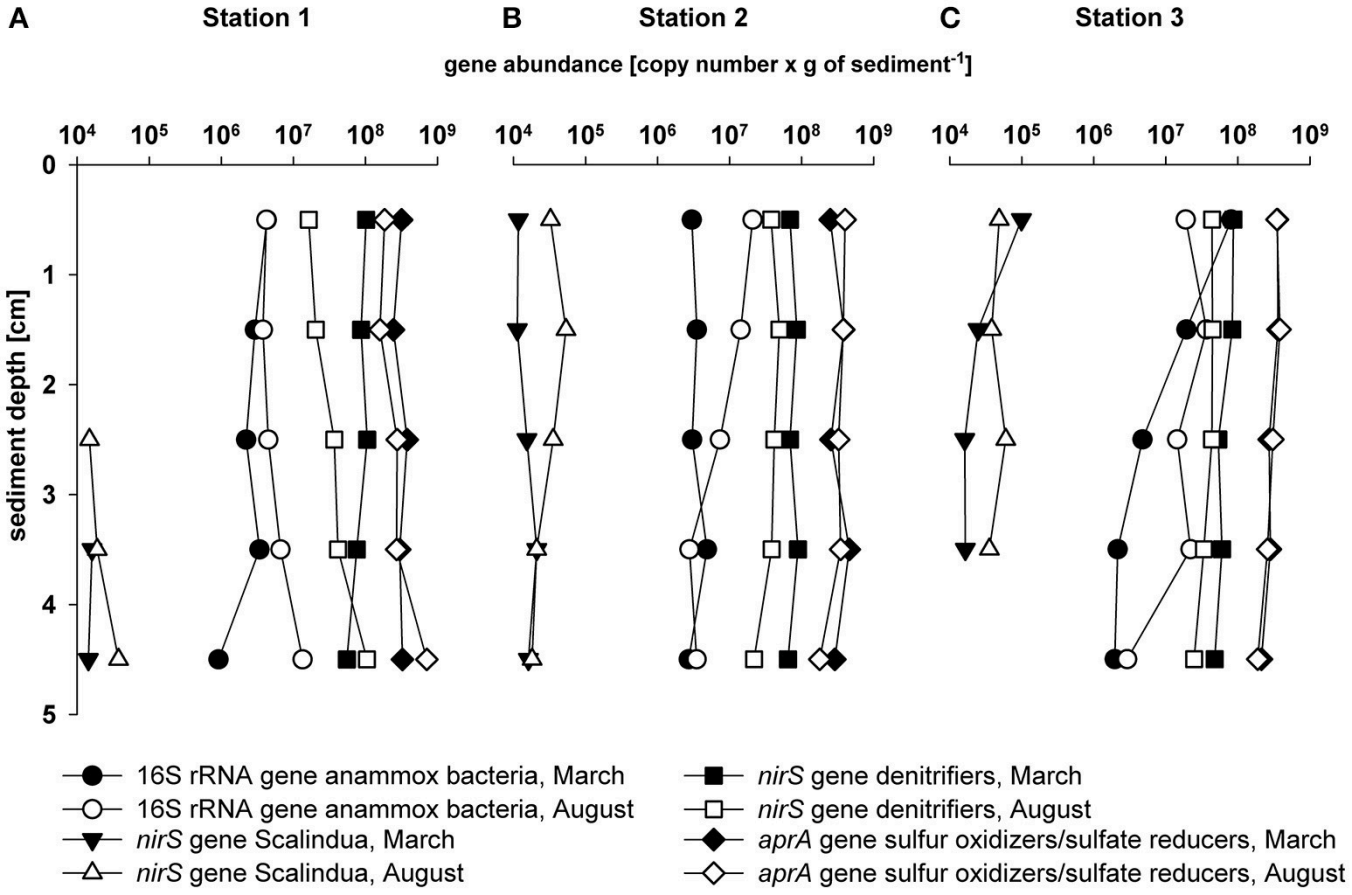
**Depth profiles of anammox bacteria 16S rRNA gene abundance [copies g^**−1**^] (circle); anammox bacteria ***nirS*** gene abundance [copies g^**−1**^] (triangle), denitrifying bacteria ***nirS*** gene abundance [copies g^**−1**^] (square); SOB and SRB ***aprA*** gene abundance [copies g^**−1**^] (diamond); (A) Station 1; (B) Station 2; (C) Station 3; black symbol: March; white/gray symbol: August**.

The abundance and distribution of *nirS*-type denitrifiers was estimated through quantification of the *nirS* gene copy number in the upper 5 cm (1 cm resolution) of the sediments at the three sampling sites in March and August (Figure 2). The *nirS* gene abundance was relatively stable with depth with slightly higher values in station S1 (6.4 × 10^7^ copies g^−1^ on average) compared to stations S2 and S3 (5.7 × 10^7^ and 5.2 × 10^7^ copies g^−1^ on average, respectively). Overall, *nirS* gene abundance was slightly higher in March compared to August in S2 and S3, and this difference was especially evident in station S1.

To estimate the potential transcriptional activity of *nirS*-type denitrifiers, *nirS* transcripts (mRNA copy numbers) were quantified (data reported in Table S3) and the RNA:DNA ratio was calculated. In station S1, transcripts were only detectable in March in the upper 2 cm of the sediment, 5.6 × 10^3^ copies g^−1^on average, whereas in August, *nirS* gene transcripts could only be detected within the 2–3 cm depth layer (10^2^ copies g^−1^). In station S2, *nirS* gene transcripts were only detectable in March in the upper 3 cm and numbers varied between 1.9 × 10^2^ and 1.5 × 10^3^ copies g^−1^ with the highest value within the 1–2 cm zone. In station S3, the *nirS* gene transcripts could be detected in March in the upper cm of the sediment (1.5 × 10^2^ copies g^−1^) and in August for 1–2 cm and for 3–4 cm sediment (1.1 × 10^2^ and 1.8 × 10^3^ copies g^−1^, respectively; Table S3). The ratio of *nirS* gene and transcript copies (RNA:DNA ratio) was ≤0.00013 in all stations in March and August.

### Diversity, abundance, and potential transcriptional activity of anammox bacteria

**Figure 3:**
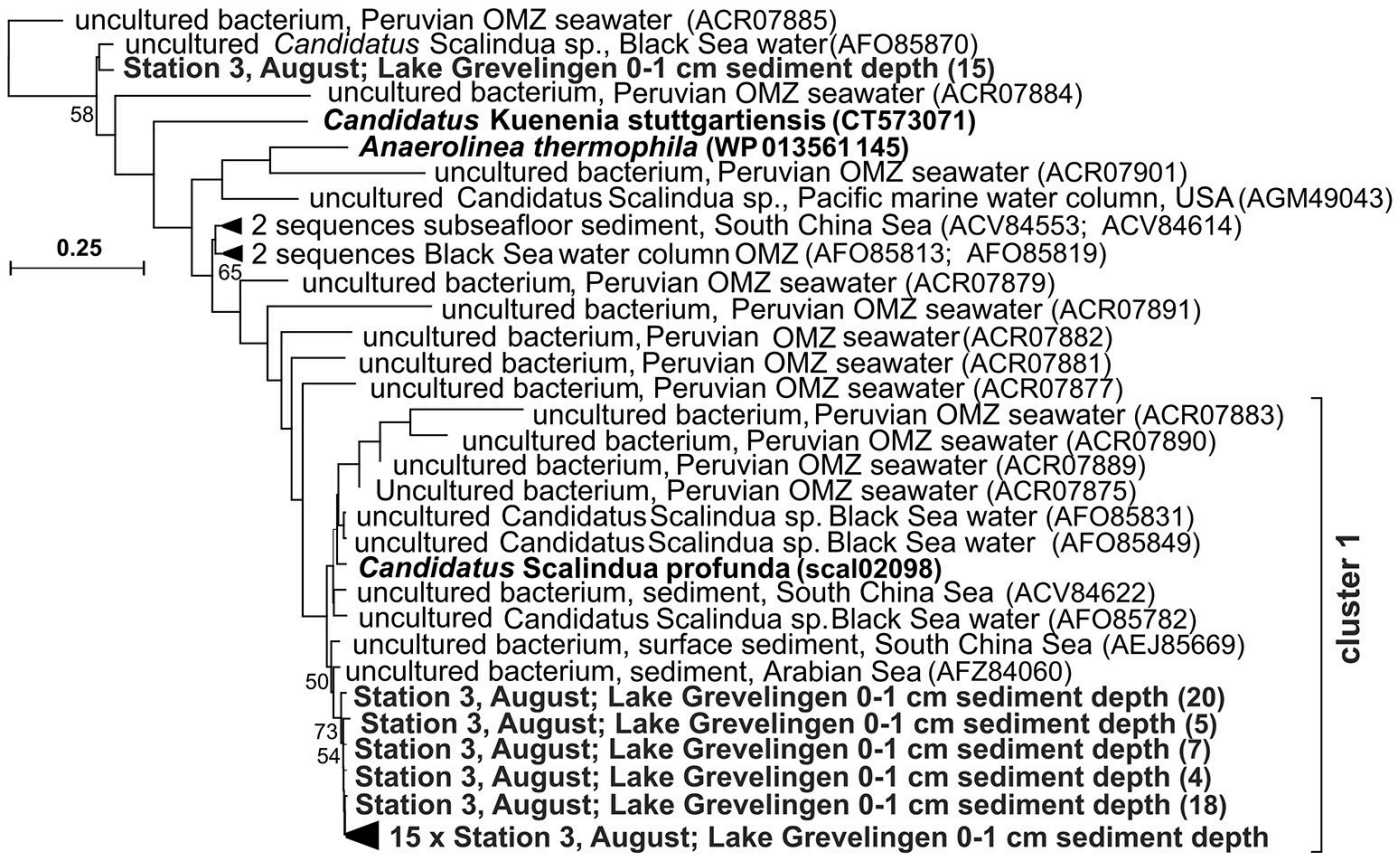
**Phylogenetic tree of partial ***nirS*** gene sequences of anammox bacteria retrieved in this study and closest relatives (S3; 0 and 1 cm Lake Grevelingen sediment; bold: sequences retrieved in March and sequences of known relatives); the scale bar indicates 25% sequence divergence**.

Most of the anammox bacterial *nirS* gene sequences obtained in this study (20 sequences out of 21) were part of one cluster (cluster 1, Figure 3) closely related to *nirS* sequences of “*Candidatus* Scalindua profunda” (van de Vossenberg et al., 2013), and of uncultured bacteria obtained from continental margin sediment of the Arabian Sea (Sokoll et al., 2012) and surface sediments of the South China Sea (Li et al., 2013).

The diversity of the 16S rRNA gene sequences of anammox bacteria (Figure S3) obtained from surface sediments (0–1 cm) revealed that most of the sequences (21 sequences derived from PCR plus cloning, and 23 gene sequences obtained from the qPCR assay and further cloning to check the specificity of the qPCR assay) were closely related to “*Candidatus* Scalindua brodae” and “*Candidatus* Scalindua marina” obtained from surface sediments of the Gullmar Fjord (Brandsma et al., 2011), and to sequences detected in OMZ waters of the Arabian Sea, Peru, and Namibia (Woebken et al., 2008) and Peruvian coastal margin (Henn, unpublished). Two 16S rRNA sequences were more distantly related to the other sequences, and were closely related to the 16S rRNA sequence of “*Candidatus* Scalindua wagneri” obtained from a bioreactor (Woebken et al., 2008; Figure S3).

We also quantified the C_20_-[3]-ladderane monoether-PC (for 0–5 cm sediment depth) and ladderane core lipids (for 0–1 and 4–5 cm sediment depth; Tables S4, S5) as markers for the presence of anammox bacteria. Abundance of PC-monoether ladderane ranged between 0.9 and 20.4 ng g^−1^ with highest values in station S1 in March (between 3.4–20.4 and 2.4–7 ng g^−1^, respectively). PC-monoether ladderane values were relatively constant with depth in August in all stations (between 0.9 and 7 ng g^−1^; Table S4). On the other hand, PC-monoether ladderane abundance was always higher in March in the upper 2 cm of the sediment and decreased four-fold between 2 and 5 cm in all stations (on average from 10.7 to 2.5 ng g^−1^). The summed concentration of the ladderane fatty acids was on average lowest in station S1 (13 ng g^−1^), with slightly higher average values in S2 (27 ng g^−1^), and the highest average values in S3 (41 ng g^−1^; Table S5). The summed ladderane fatty acid concentrations were comparable to the abundance of anammox bacteria determined by the 16S rRNA gene quantification in the first centimeter of the sediment obtained in different stations and seasons, whereas the PC-monoether ladderane revealed a different trend compared to the 16S rRNA gene abundance, i.e., was more abundant in March compared to August (Table S4).

The abundance of anammox bacteria was determined by the quantification of the 16S rRNA gene copy number of anammox bacteria as well as of the *nirS* gene copy number of members of the genus “*Candidatus* Scalindua” (Figure 2; Strous et al., 2006; Li et al., 2011). Copy numbers of the anammox bacteria 16S rRNA gene ranged between 9.5 × 10^5^ and 8.1 × 10^7^ copies g^−1^ with highest values in S3 (between 2 × 10^6^ and 8.1 × 10^7^copies g^−1^). The abundance of the anammox bacterial 16S rRNA gene was slightly higher in August compared to March in all stations. The “*Candidatus* Scalindua” *nirS* gene abundance followed the same depth trend but values were 2–3 orders of magnitude lower (between 1.1 × 10^4^ and 9.9 × 10^4^ copies g^−1^) compared to the anammox bacteria 16S rRNA gene copy numbers.

The anammox bacterial 16S rRNA transcript, used as a proxy for the potential transcriptional activity, varied between 1.3 × 10^3^–5.1 × 10^6^ copies g^−1^ of sediment (Table S3). The anammox 16S rRNA transcript copy number varied slightly and reached highest values in S2 and S3 in August between 2 and 4 cm sediment depth (2.7 × 10^6^ copies g^−1^ on average). The ratio between 16S rRNA gene and transcript (RNA:DNA ratio) was ≤0.32 in all stations in March and August. Anammox bacteria *nirS* transcript copies were below detection level of the qPCR assay.

### Diversity, abundance, and potential transcriptional activity of bacteria involved in sulfur cycling

**Figure 4:**
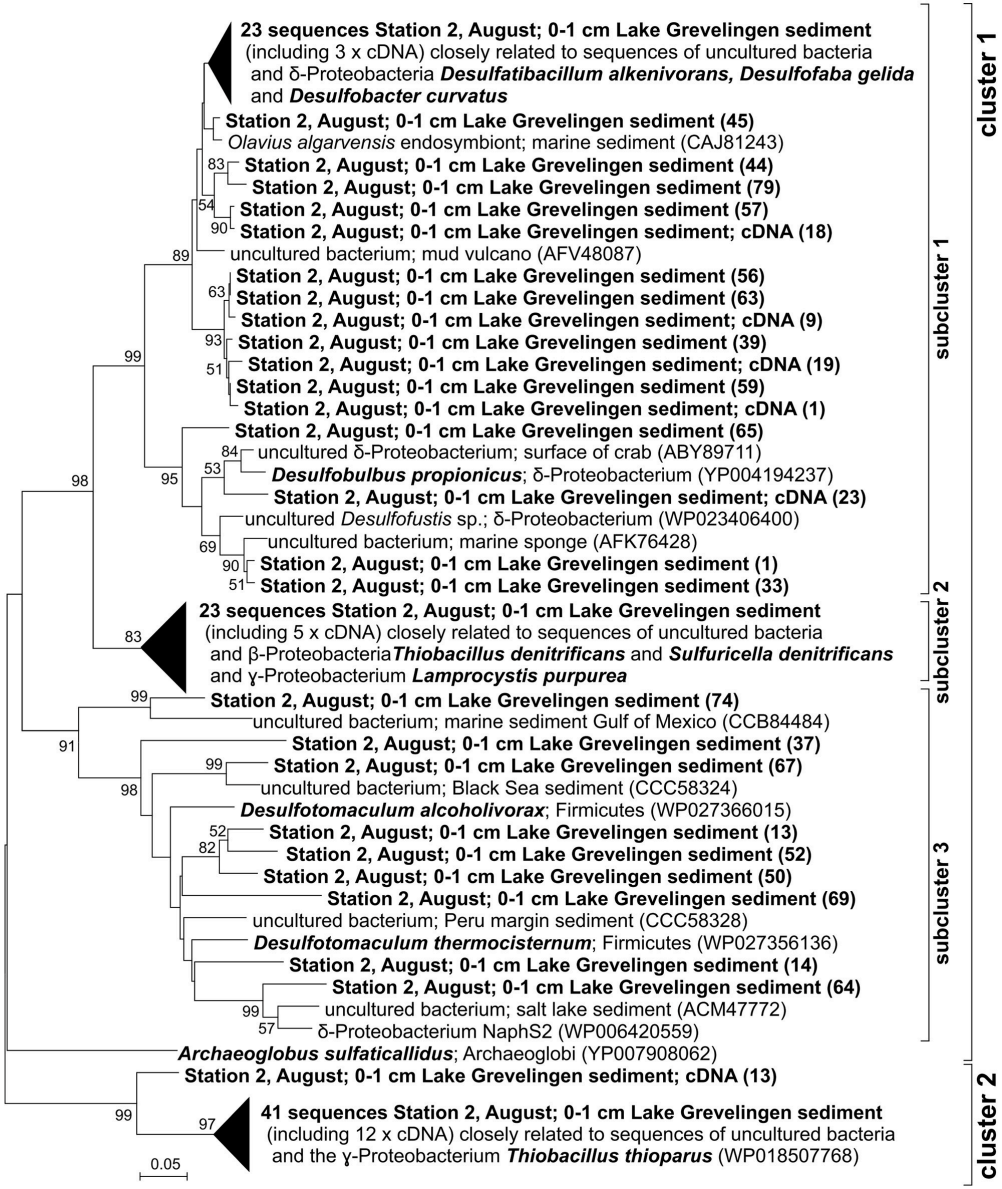
**Phylogenetic tree of partial ***aprA*** gene sequences of sulfur-oxidizing and sulfate-reducing bacteria (SOB and SRB) retrieved in this study (86 DNA sequences recovered by amplification (PCR) and 21 cDNA sequences recovered by amplification (qPCR) and cloning from sediments (0–1 cm) of station S2 in August) and closest relatives (bold: our sequences and closest known relatives); the scale bar indicates 25% sequence divergence**.

Here, we focused on bacteria involved in sulfur cycling, performing dissimilatory sulfur oxidation or sulfate reduction. The dissimilatory APS reductase encoded by the *aprA* gene is operating in SRB, converting APS to adenosine monophosphate (AMP) and sulfite (${\text{SO}}_{3}^{2-}$), as well as in SOB, operating in reverse direction, oxidizing sulfite to APS (Meyer and Kuever, 2007b). The diversity of the microorganisms harboring the *aprA* gene was evaluated in the first centimeter of the sediment core at S2 in August as the observation of *Beggiatoa* mats on top indicated the occurrence of sulfide oxidation at this station. The protein sequences (86 sequences PCR + cloning) coded by the *aprA* gene grouped in two clusters [Figure 4; cluster I (66%) and II (34%)]. Within cluster I, sequences clustered in three distinct subclusters. Sequences of the *aprA* gene included in subcluster 1.1 (36%) were affiliated to heterotrophic SRB of the δ-proteobacteria class (e.g., *Desulfosarcina* sp., *Desulfofaba gelida, Desulfobulbus propionicus*) and to *aprA* gene sequences of uncultured bacteria found in environments such as in Black Sea sediments and associated with benthic organisms (Ruehland et al., 2008; Blazejak and Schippers, 2011). In subcluster 1.2 (21%), sequences were closely affiliated to β- and γ-proteobacteria involved in autotrophic sulfur-dependent denitrification (*Thiobacillus denitrificans* and *Sulfuricella denitrificans*) or in phototrophic sulfur oxidation (*Lamprocystis purpurea*) and to the *aprA* gene sequence of an uncultured bacterium associated with the sea urchin *Asterechinus elegans* (Quast et al., 2013). Sequences clustering in subcluster 1.3 (9%) were affiliated with *aprA* gene sequences of bacteria of the phylum Firmicutes (i.e., *Desulfotomaculum* sp.) known to perform heterotrophic sulfate reduction, and to sequences of uncultured bacteria obtained in various sediments such as hydrothermal seep sediments, Peru margin sediments, and salt lake sediments (Meyer and Kuever, 2007c; Blazejak and Schippers, 2011; Kleindienst et al., 2012). Cluster II contained sequences (34%) closely related to obligately chemolithoautotrophic members of the β-proteobacteria class (*Thiobacillus thioparus*) able to perform nitrate reduction to nitrite with thiocyanate, and to *aprA* gene sequences of uncultured bacteria retrieved in salt lake sediments or associated with benthic organisms (Becker et al., 2009). In addition, to assess the specificity of the *aprA* gene qPCR assay, *aprA* gene transcripts (cDNA of *aprA* mRNA) generated during the qPCR assay (21 sequences in total) were cloned, sequenced, and added to the *aprA* phylogenetic tree, where they were classified in the clusters previously described (Figure 4).

The abundance and the potential activity of SOB including sulfide-dependent denitrifiers and SRB were determined by the quantification of the *aprA* gene and its gene transcripts (Figure 2, Table S3). The copy number of the *aprA* gene was relatively constant with varying sediment depth and season Gene copy numbers of the *aprA* gene were of the same order of magnitude as observed for the denitrifiers *nirS* (on average 3.1 × 10^8^ copies g^−1^) and reached highest values in S2 in March and August (on average 3.3 × 10^8^ copies g^−1^). At station S1, the *aprA* gene transcript (Table S3) was detectable at 2–3 cm sediment depth in March (5.4 × 10^2^ copies g^−1^ on average), whereas in August the *nirS* transcript was detectable at 0–1 cm (3.8 × 10^3^ copies g^−1^) and 2–4 cm (1.8 × 10^4^ copies g^−1^ on average) sediment depth. In S2 and S3, *aprA* gene transcripts were detectable at 1–4 cm sediment depth in March and August (on average 4.7 × 10^3^ copies g^−1^ in March and 4.4 × 10^3^ copies g^−1^ in August). The ratio between *aprA* gene and transcript (RNA:DNA ratio) was ≤0.000088 in all stations in March and August.

## Discussion

The phylogenetic analysis of the *nirS* gene sequences amplified from Lake Grevelingen sediments revealed a substantial diversity of autotrophic and heterotrophic denitrifiers (Figure 1). Most of the sequences obtained in this study were affiliated to α-, β-, and γ-proteobacteria able to perform denitrification coupled to the oxidation of reduced sulfur compounds. Sequences of the *nirS* gene (~30%) and the *aprA* gene (21%; Figure 4) were closely related to sequences of *Thiobacillus denitrificans*, able to couple the oxidation of inorganic sulfur species such as sulfide with denitrification (Shao et al., 2010). Sulfide-dependent denitrifiers couple nitrogen and sulfur cycling and their presence point to an important denitrification potential in these sediments. In order to determine differences in abundance, and therefore of denitrification potential, between sediments and with depth, in our study we quantified the abundance of the *nirS* gene. The *nirS* gene abundance was relatively constant with increasing sediment depth, suggesting a stable community of heterotrophic and autotrophic denitrifying bacteria in Lake Grevelingen sediments. *NirS* gene abundance (in the order of 10^7^ copies g^−1^) was slightly higher in March in all three stations, but the difference was especially notable in station S1, most likely explained by the lower free sulfide concentrations in March compared to August (Table 1). Lower sulfide concentrations in March could favor the proliferation of heterotrophic denitrifiers not coupled with sulfide oxidation as sulfide has been seen to impair denitrification by inhibition of NO and N_2_O reductases (Sørensen et al., 1980). In addition, the *nirS* gene abundance (Figure 2) determined in Lake Grevelingen sediments (between 10^7^ and 10^8^ copies g^−1^) was one to three orders of magnitude higher compared to the values previously detected in other marine sediments such as in sediments of open estuaries with permanently oxygenated bottom water (10^4^–10^7^ copies g^−1^ on average; Smith et al., 2007; Zhang et al., 2014). The *nirS* gene abundance has previously been shown to correlate positively with potential denitrification rates (Mosier and Francis, 2010), and therefore, we can conclude that the sediments of the marine Lake Grevelingen harbor a stable community of heterotrophic and autotrophic denitrifiers, which can be actively involved in the denitrification process independent of season and sediment depth.

Besides quantifying the *nirS* gene as an indicator of the denitrification potential of these sediments, we also estimated the presence of *nirS* gene transcripts as a measure of potential activity (Table S3). Unfortunately, there is a lack of *in situ* studies correlating *nirS* gene expression with denitrification rates, and some studies have even shown a lack of direct relationship between *nirS* gene expression and modeled rates of denitrification in marine surface sediments (Bowen et al., 2014). On the other hand, other studies have observed a correlation between a decrease of *nirS* gene transcripts and reduction of denitrification rates together with declining concentrations of nitrate in estuarine sediment (Dong et al., 2000, 2002; Smith et al., 2007), which would justify the use of *nirS* gene transcripts as a proxy for potential denitrification performed by *nirS*-type denitrifiers. In this study, the copy number of denitrifier *nirS* transcripts was two to three orders of magnitude lower compared to values previously reported for estuarine sediments (Smith et al., 2007), suggesting a low denitrification rate in surface sediments of all stations in March and in deeper sediment layers in August. Gene expression of the *nirS* gene was detected in surface sediments in March (all stations) and in deeper sediment layers in August (stations S1 and S3), which is most likely explained by the availability of nitrate/nitrite in the water-sediment interface (bottom water).

Apart from *nirS*-containing denitrifiers, also anammox bacteria were detected in Lake Grevelingen sediments by applying both DNA and lipid-based biomarkers. The phylogenetic analysis of the anammox 16S rRNA gene amplified from the sediments pointed to an anammox community dominated by “*Candidatus* Scalindua” (Figure S3). In order to estimate the abundance of anammox bacteria in the sediments and their potential role in the nitrogen cycle in this system, we estimated the abundance of the “*Candidatus* Scalindua” *nirS* gene. In contrast to *nirS*-type denitrifiers, anammox bacteria showed a clear seasonal contrast in their *nirS* gene abundance with higher copy numbers in August (summer hypoxia) compared to March (Figure 2). However, this seasonal trend was not reflected in the abundance of the anammox bacteria lipid biomarker PC-monoether ladderane lipid (Table S4). Previous studies have suggested that this biomarker lipid could be partly preserved in the sediment due to a relatively low turnover rate (Brandsma et al., 2011; Bale et al., 2014; Lipsewers et al., 2014). On the other hand, the concentration of ladderane lipid fatty acids (Table S5) correlated with the anammox bacteria abundance given by 16S rRNA gene quantification, i.e., with the lowest values in station S1 and highest values in station S3. This suggests that the abundance of ladderane lipid fatty acids could be interpreted as a proxy for anammox bacteria abundance together with specific gene quantification. It is also worth noticing that a difference of three to four orders of magnitude was detected between abundances of anammox 16S rRNA gene and the “*Candidatus* Scalindua” *nirS* gene (Figure 2). This discordance between anammox 16S rRNA genes and functional genes has been previously observed and is possibly linked to primer biases attributed to the anammox bacteria 16S rRNA gene primers that would amplify 16S rRNA gene fragments of bacteria other than anammox bacteria (Li et al., 2010; Harhangi et al., 2012; Bale et al., 2014; Lipsewers et al., 2014). However, in our study we have ruled out the possibility of the anammox bacteria 16S rRNA gene primers amplifying bacteria other than anammox by cloning and sequencing of the product generated during the qPCR reaction as shown in Figure S3. Therefore, further studies should address the causes of this discordance.

Factors other than hypoxia and sulfide concentration could have also contributed to the differences in abundance and activity of anammox bacteria. For example, both the anammox bacteria 16S rRNA and *nirS* gene abundances were higher in August in comparison to March (Figure 2). This may indicate that anammox bacteria are more abundant at higher temperatures (15°C), which is the anammox bacteria temperature optimum in temperate shelf sediments (Dalsgaard et al., 2005). This seasonality of anammox bacteria abundance has been reported before in sandy and as well in muddy, organic rich sediments of the North Sea (Bale et al., 2014; Lipsewers et al., 2014). Additionally, OPD in the sediments decreased and the sediment even became entirely anoxic during summer stratification (Table 1), which would also favor the anaerobic metabolism of anammox bacteria (Dalsgaard and Thamdrup, 2002). Anammox bacterial abundance was highest in station S3, which could also be related to elevated bioturbation activity that was observed in this station, as bioturbation and mixing extends the area of nitrate reduction, which might fuel the anammox process (Meyer et al., 2005; Laverock et al., 2013). Another factor determining anammox and denitrification pathways in sediments is the organic carbon content. Anammox and denitrification have been suggested to be more important nitrogen removal pathways in sediments of low carbon input compared to sulfide-dependent denitrification which seem to be more important in sulfidic sediments with high carbon input (Burgin and Hamilton, 2007). The TOC content in Lake Grevelingen sediments is high, i.e., in the order of 2.5–4.5% (Malkin et al., 2014), which is hence consistent with the low activity of anammox bacteria. Moreover, in station S1, fresh organic-rich sediment rapidly accumulates (>2 cm yr^−1^; Malkin et al., 2014), which might inhibit the activity of anammox bacteria and denitrifying bacteria.

Overall, the *nirS* gene abundance of denitrifiers was three to four orders of magnitude higher compared to the anammox bacteria “Scalindua” *nirS* gene values, suggesting that *nirS*-type denitrifiers have a more prominent role in the overall denitrification activity in Lake Grevelingen sediments in comparison with anammox bacteria (Figure 2). This is also supported by the anammox bacteria *nirS* gene transcript abundance, which remained below detection limit. On the other hand, anammox bacteria 16S rRNA gene transcripts were detected throughout the sediment (Table S3). A recent study by Bale et al. (2014) observed a good correlation between the 16S rRNA gene abundance, 16S rRNA gene transcript abundance and anammox rates in North Sea sediments, which suggests that the transcriptional activity of anammox bacteria 16S rRNA gene is a suitable proxy of anammox bacteria activity. However, the anammox bacteria 16S rRNA gene RNA:DNA ratio was low (0.01–1) compared to the values previously reported in sediments of the southern North Sea (1.6–34.6; Lipsewers et al., 2014), further supporting a low anammox activity in the Lake Grevelingen sediments.

Although denitrification was more relevant than the anammox process as suggested by the gene abundance and potential activity (Figure 2, Table S3), the measured denitrification rates (between 36 and 96 μM m^−2^ d^−1^; D. Seitaj, personal communication) in Lake Grevelingen are low in comparison with other marine sediments. For example, denitrification rates were reported to vary between 30 and 270 μM m^−2^ d^−1^ in marine Arctic sediments (Rysgaard et al., 2004), while denitrification rates of 270 ± 30μmol N_2_ m^−2^ d^−1^ were measured in sediments of the Lower St. Lawrence Estuary (Crowe et al., 2012). The lower denitrification and anammox potential in Lake Grevelingen sediments could be explained by the effect of high concentrations of sulfide (potentially inhibiting both heterotrophic denitrification and anammox). In our study, the abundance of *nirS*-type denitrifiers did not vary substantially for the different stations, however, the highest anammox bacterial abundance was observed in station S3 where lowest sulfide concentration was reported (Figure 2, Table 1). Besides, these sediments harbor an important population of sulfide-dependent denitrifiers (as indicated by *aprA* gene values comparable to those found in sediments of the Black Sea; Blazejak and Schippers, 2011), which could alleviate the inhibitory effect of sulfide on other microbial groups such as anammox bacteria. For example, a study by Russ et al. (2014) observed the coexistence and interaction of sulfide-dependent denitrifying and anammox bacteria in a co-culture in which anammox bacteria remained active. Also a study by Wenk et al. (2013) provided evidence for the coexistence of anammox bacteria and sulfide-dependent denitrifiers in the stratified water column of Lake Lugano, and reported that the addition of sulfide in incubation studies enhanced both processes. They speculated that anammox bacteria in this system would rely on nitrite released as intermediate during sulfide-dependent denitrification and that they would overcome inhibiting or toxic effects of sulfide by creating sulfide-free microenvironments in aggregates as previously proposed by Wenk et al. (2013). However, in the case of Lake Grevelingen sediments the potential detoxification of sulfide and a source of nitrite by sulfide-dependent denitrifiers did not translate in significant anammox potential.

Another explanation for the low denitrification potential of these sediments can be found in the interactions of anammox and heterotrophic denitrifiers with sulfur-oxidizers also involved in the nitrogen cycle. For example in Lake Grevelingen, cable bacteria have been detected in stations S1 and S3 in March whereas station S2 was dominated by Beggiatoaceae down to ~3 cm, but both groups were hardly detectable in subsurface sediments (0.5 cm downwards) during summer hypoxia (Seitaj et al., 2015; Seitaj, unpublished data). In fact, the analysis of *aprA* gene of SOB/SRB in our study showed that up to 36% of the *aprA* gene sequences were closely related to the *aprA* sequences of *Desulfobulbus propionicus* (Figure 4), which has been identified as closest known relative of cable bacteria (Pfeffer et al., 2012). Cable bacteria perform a novel “electrogenic” form of sulfur oxidation, in which the oxidation of the electron donor and the reduction of the electron acceptor are separated over centimeter-scale distances, and the necessary redox coupling is ensured by long-distance electron transport (Nielsen et al., 2010; Pfeffer et al., 2012). Cable bacteria use oxygen as terminal electron acceptor (Nielsen et al., 2010; Meysman et al., 2015) and recent studies also indicate that nitrate (Marzocchi et al., 2014) and nitrite (Risgaard-Petersen et al., 2014) can be utilized, suggesting that cable bacteria can also play a role in bioavailable nitrogen removal. In addition, marine *Beggiatoa* spp. couple the oxidation of sulfide to nitrate reduction resulting in N_2_ and/or ${\text{NH}}_{4}^{+}$ (Mußmann et al., 2003). Previous studies have observed that both denitrification and anammox in anoxic sediments can be supported by intracellular nitrate transport performed by sulfide-oxidizing bacteria like *Thioploca* and *Beggiatoa* (Mußmann et al., 2007; Jørgensen, 2010; Prokopenko et al., 2013) down to deeper sediment layers and possibly supplying anammox bacteria with nitrite and/or ammonia produced by DNRA (Teske and Nelson, 2006; Prokopenko et al., 2011). The *nrfA* gene, encoding a nitrite reductase catalyzing the conversion of nitrite to ammonia (Smith et al., 2007), could not be detected in our sediment samples (data not shown) by using general *nrfA* primers (Mohan et al., 2004), however we cannot rule out completely the presence of microorganisms performing DNRA as the primers used could be not the most appropriate ones for this system. Therefore, the presence of sulfide-dependent denitrifiers (such as *Thiobacillus denitrificans*), cable bacteria and *Beggiatoa*-like bacteria present in Lake Grevelingen sediments could potentially support denitrification and anammox processes in March. However, the nitrate and nitrite concentrations in the bottom water in March were reported to be relatively low (28 and 0.7 μM on average, respectively, Figure S1). The limitation of nitrite and nitrate is expected to induce a strong competition for nitrate, nitrite and sulfide with Beggiatoaceae, cable bacteria and other nitrate-reducing bacteria (e.g., *Thiobacillus thioparus*), which may explain the limited denitrification potential observed in the Lake Grevelingen sediments.

## Conclusion

Our study has unraveled the coexistence and potential activity of heterotrophic and autotrophic denitrifiers, anammox bacteria as well as SOB/SRB in seasonally hypoxic and sulfidic sediments of Lake Grevelingen. Our starting hypothesis was that the abundance and activity of denitrifiers and anammox bacteria would decrease upon increase of the sulfide concentration found in the sediments during the summer hypoxia. However, *nirS*-type heterotrophic denitrifiers were a stable community regardless of changes in oxygen and sulfide concentrations in different seasons and with a similar abundance to that detected in other sediments not exposed to high sulfide concentrations.

Besides, *nirS*-type denitrifiers outnumbered anammox bacteria leading to the conclusion that anammox does not contribute significantly to the N_2_ removal process in Lake Grevelingen sediments. Apart from that, the anammox bacteria population seemed to be affected by the physicochemical changes between seasons. For example, their abundance and activity was higher in lower sulfide concentrations and low carbon input, also supporting a possible inhibition of anammox bacteria by sulfide. The sulfide-dependent denitrifiers in Lake Grevelingen sediments have proven to be abundant and expected to contribute significantly to the N_2_-removal in these sediments. Their activity of sulfide oxidation is intuitively expected to reduce the concentration of sulfide, which is in turn toxic for organoheterotrophic denitrifiers and anammox bacteria. However, in this system the detoxification mediated by sulfide-dependent denitrifiers is either not sufficient or other factors are contributing to the low denitrification potential observed in the sediments of Lake Grevelingen.

Recent studies have also reported the presence of cable bacteria and sulfide-oxidizers of the Beggiatoaceae family in the Lake Grevelingen sediments. Both denitrifiers and anammox bacteria activity could be inhibited by the competition with cable bacteria and Beggiatoaceae for electron donors and acceptors (such as sulfide, nitrate, and nitrite) before summer hypoxia (March). During summer hypoxia (August), sulfide inhibition and low nitrate and nitrite concentrations seem to limit the activity of heterotrophic and autotrophic (sulfide-dependent) denitrifiers and anammox bacteria. Further studies also involving denitrification rate determinations will be required to further assess the effects of hypoxia and high sulfide concentrations in the sediments of Lake Grevelingen.

## Author contributions

YL performed the sampling, contributed to the experimental design, data analysis, and writing of the manuscript. EH contributed to the data analysis and writing. FM contributed to the sampling and writing. JS contributed to the data analysis and writing. LV contributed to the sampling, experimental design, data analysis, and writing of the manuscript.

### Conflict of interest statement

The authors declare that the research was conducted in the absence of any commercial or financial relationships that could be construed as a potential conflict of interest.
