# Supplementary material for: Abundance and Diversity of Denitrifying and Anammox Bacteria in Seasonally Hypoxic and Sulfidic Sediments of the Saline Lake Grevelingen
Source: Front Microbiol. 2016 Oct 20;7:1661. doi: 10.3389/fmicb.2016.01661 (PMC5071380; doi:10.3389/fmicb.2016.01661)
Supplement: Supplementary file 1 [file Table1.PDF]

1 Supplementary Tables

Table S1: Primer pairs described in the text, PCR conditions and amplicon size used in this study.

Assay	Target	Primer pair	Tm [°C]	Amplicon size [b]	Reference
qPCR + PCR/cloning	Anammox bacteria 16S rRNA gene	Brod541F (5'-GAGCACGTAGGTGGGTTTGT-3')<br>Amx820R (5'-AAAACCCCTCTACTTAGTGCCC-3')	qPCR 59/ PCR 58	279	1
qPCR + PCR/cloning	*Scalindua* sp. *nirS* gene	Scnir372F (5'-TGTAGCCAGCATTGTAGCGT-3')<br>Scnir845R (5'-TCAAGCCAGACCCATTTGCT-3')	qPCR/ PCR 57	474	2
qPCR + PCR/cloning	Denitrifying bacteria *nirS* gene	nirS1F (5'-CCTAYTGGCCGCCRCART-3')<br>nirS3R (5'-GCCGCCGTCRTGVAGGAA-3')	qPCR/ PCR 60	222	3
qPCR+ cloning	Sulfide oxidizing bacteria *aprA* gene	Apr1F (5'TGGCAGATCATGATYMAYGG-3')<br>Apr5R (5'-GCGCCAACYGGRCCRTA-3')	qPCR/ PCR 56	359	4

PCR conditions: 95°C 5 min; 40 × [95°C 1 min, Tm 40 s, 72°C 1 min]; 72°C 5 min. qPCR conditions: 95°C 4 min; 40 × [95°C 30 s, Tm 40 s, 72°C 30 s]; 80°C 25 s.

1. Li *et al*., 2010
2. Lam *et al*., 2009
3. Bracker *et al*., 1998
4. Meyer & Kuever, 2007
