# Supplementary material for: Abundance and Diversity of Denitrifying and Anammox Bacteria in Seasonally Hypoxic and Sulfidic Sediments of the Saline Lake Grevelingen
Source: Front Microbiol. 2016 Oct 20;7:1661. doi: 10.3389/fmicb.2016.01661 (PMC5071380; doi:10.3389/fmicb.2016.01661)
Supplement: Supplementary file 2 [file Table2.PDF]

Table S2: Total organic carbon (TOC) of the sediment after acidification of freeze dried sediment samples (1 cm resolution) of all stations in March and in August.

Station	Sediment depth [cm]	TOC [%]	
		March	August
1	0–1	2.86	1.81
	1–2	3.67	2.47
	2–3	3.83	3.52
	3–4	3.98	3.11
	4–5	4.28	3.66
2	0–1	3.11	2.38
	1–2	3.62	3.29
	2–3	3.77	3.24
	3–4	3.49	3.5
	4–5	3.85	3.52
3	0–1	2.87	3.04
	1–2	4.02	3.9
	2–3	3.89	3.56
	3–4	4.01	4.39
	4–5	3.63	3.73
