# Supplementary material for: Abundance and Diversity of Denitrifying and Anammox Bacteria in Seasonally Hypoxic and Sulfidic Sediments of the Saline Lake Grevelingen
Source: Front Microbiol. 2016 Oct 20;7:1661. doi: 10.3389/fmicb.2016.01661 (PMC5071380; doi:10.3389/fmicb.2016.01661)
Supplement: Supplementary file 3 [file Table3.PDF]

Table S3: Transcript (cDNA; mRNA copy) abundance detected in sediments of all stations in March and August.

Station	Sediment depth [cm]	*nirS* gene denitrifying bacteria cDNA [copy g^{-1}]		16S rRNA gene anammox bacteria cDNA [copy g^{-1}]		*aprA* gene SOB and SRB cDNA [copy g^{-1}]	
		March	August	March	August	March	August
1	0–1	1.8×10^2	n.d.	1.9×10^5	8.1×10^5	n.d.	3.8×10^3
	1–2	1.1×10^4	n.d.	1.8×10^5	1.3×10^3	n.d.	n.d.
	2–3	n.d.	1.0×10^2	1.0×10^5	2.8×10^5	5.4×10^2	2.5×10^4
	3–4	n.d.	n.d.	6.5×10^4	6.9×10^5	n.d.	1.1×10^4
	4–5	n.d.	n.d.	2.5×10^4	2.3×10^5	n.d.	n.d.
2	0–1	1.9×10^2	n.d.	8.5×10^4	3.0×10^5	n.d.	n.d.
	1–2	1.5×10^3	n.d.	6.0×10^5	1.1×10^6	7.0×10^3	7.4×10^2
	2–3	6.7×10^2	n.d.	9.6×10^5	1.7×10^6	1.4×10^4	3.4×10^3
	3–4	n.d.	n.d.	1.9×10^5	1.3×10^5	1.6×10^3	5.9×10^2
	4–5	n.d.	n.d.	4.8×10^4	5.5×10^4	n.d.	n.d.
3	0–1	1.5×10^2	n.d.	4.9×10^6	2.9×10^5	n.d.	n.d.
	1–2	n.d.	1.1×10^2	3.1×10^6	5.1×10^6	3.5×10^3	1.8×10^4
	2–3	n.d.	n.d.	4.1×10^5	3.1×10^6	5.0×10^2	4.3×10^2
	3–4	n.d.	1.8×10^3	2.2×10^5	1.3×10^5	1.4×10^3	3.7×10^3
	4–5	n.d.	n.d.	8.5×10^4	3.7×10^4	n.d.	n.d.

qPCR conditions: 95°C 4 min; 40 × [95°C 30 s, Tm 40 s, 72°C 30 s]; 80°C 25 s. nd: non detectable, i.e. values were under the detection limit of the qPCR approach.
