# Supplementary material for: Abundance and Diversity of Denitrifying and Anammox Bacteria in Seasonally Hypoxic and Sulfidic Sediments of the Saline Lake Grevelingen
Source: Front Microbiol. 2016 Oct 20;7:1661. doi: 10.3389/fmicb.2016.01661 (PMC5071380; doi:10.3389/fmicb.2016.01661)
Supplement: Supplementary file 4 [file Table4.PDF]

Table S4: Results of anammox bacteria PC-monoether ladderane lipid analysis in all stations in March and August (0−5 cm sediment depth).

		PC-monoether ladderane lipid [ng g^{-1}]	
Station	Sediment depth [cm]	March	August
1	0–1	20.4	2.4
	1–2	13.3	3.4
	2–3	6.1	2.8
	3–4	3.4	7
	4–5	5	7
2	0–1	10.8	6.2
	1–2	4.7	1.9
	2–3	2	1.9
	3–4	1	1.4
	4–5	1	1.4
3	0–1	8.6	2.4
	1–2	6.1	1.2
	2–3	1.6	1.2
	3–4	1.1	0.9
	4–5	1.4	1.4
