# Supplementary material for: Abundance and Diversity of Denitrifying and Anammox Bacteria in Seasonally Hypoxic and Sulfidic Sediments of the Saline Lake Grevelingen
Source: Front Microbiol. 2016 Oct 20;7:1661. doi: 10.3389/fmicb.2016.01661 (PMC5071380; doi:10.3389/fmicb.2016.01661)
Supplement: Supplementary file 5 [file Table5.PDF]

**Table S5**: Results of anammox bacteria fatty acid methyl ester (FAME) analysis of all stations in March and August (0−1 and 4−5 cm sediment depth).

| | | | long chain FAMEs [ng $g^{-1}$] | | | | sum |
|---|---|---|---|---|---|---|---|
| **Station** | **month** | **depth** | **C20-[3]** | **C20-[5]** | **C18-[3]** | **C18-[5]** | **C20-[3]–C18-[5]** |
| Station 1 | March | 0.5 | 3.6 | 3.8 | 4.0 | 5.5 | 16.8 |
| | March | 4.5 | 3.5 | 5.4 | 2.0 | 5.0 | 15.8 |
| | August | 0.5 | 2.5 | 1.5 | 5.1 | 3.9 | 13.1 |
| | August | 4.5 | 1.8 | 1.8 | 1.4 | 2.8 | 7.8 |
| Station 2 | March | 0.5 | 6.1 | 4.7 | 6.9 | 7.8 | 25.4 |
| | March | 4.5 | 4.8 | 9.1 | 4.4 | 4.4 | 22.7 |
| | August | 0.5 | 5.3 | 5.7 | 19.3 | 8.4 | 38.7 |
| | August | 4.5 | 3.1 | 8.2 | 4.4 | 4.1 | 19.8 |
| Station 3 | March | 0.5 | 18.9 | 16.3 | 32.1 | 39.3 | 106.5 |
| | March | 4.5 | 3.6 | 7.9 | 4.0 | 4.0 | 19.5 |
| | August | 0.5 | 4.7 | 4.1 | 8.9 | 9.7 | 27.4 |
| | August | 4.5 | 2.8 | 3.2 | 1.6 | 3.0 | 10.6 |
