# Supplementary material for: Abundance and Diversity of Denitrifying and Anammox Bacteria in Seasonally Hypoxic and Sulfidic Sediments of the Saline Lake Grevelingen
Source: Front Microbiol. 2016 Oct 20;7:1661. doi: 10.3389/fmicb.2016.01661 (PMC5071380; doi:10.3389/fmicb.2016.01661)
Supplement: Supplementary file 6 [file Image1.PDF]

2. Supplementary Figures

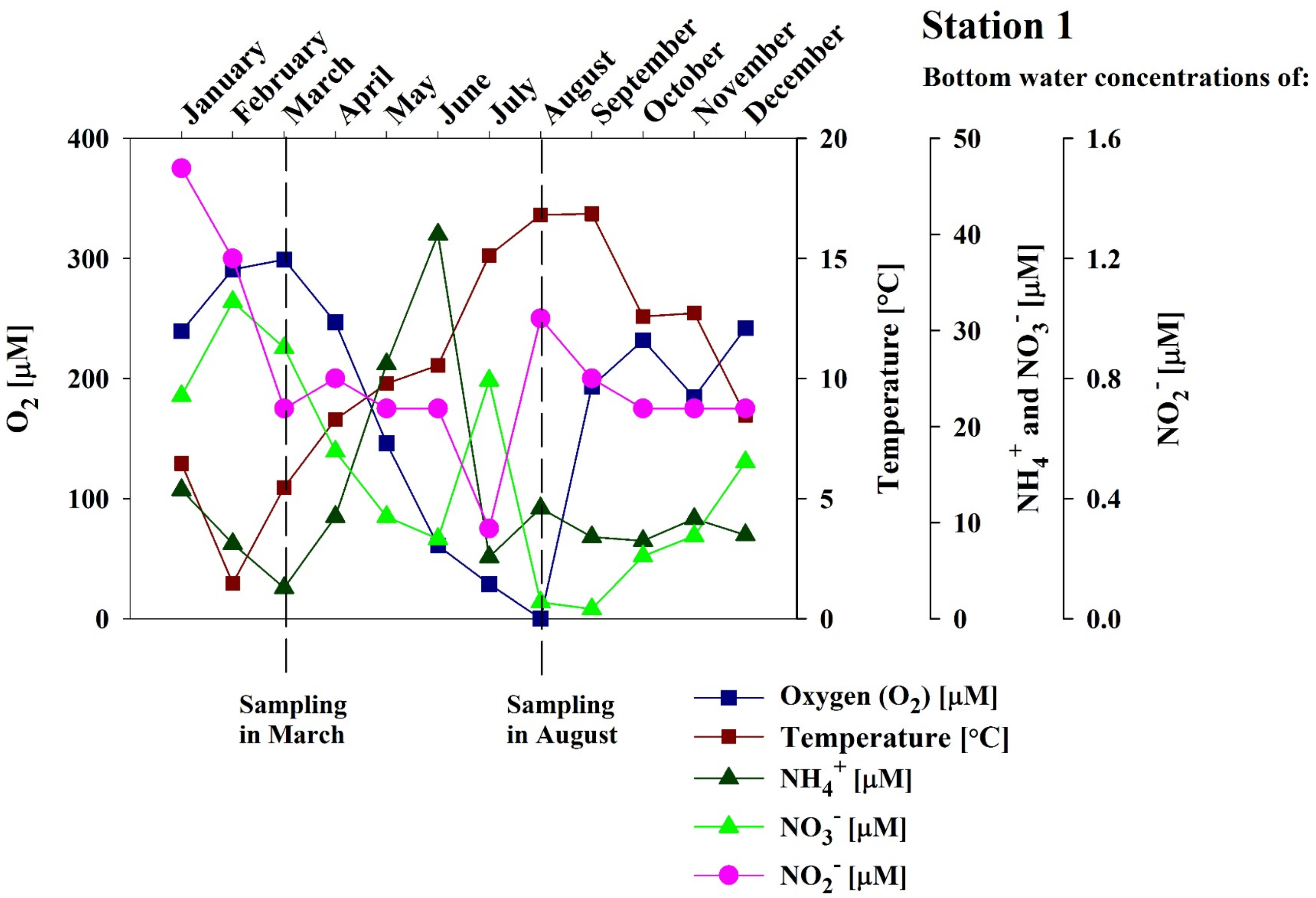

Supplementary Figure 1. Bottom water temperature [°C] (red square) and concentrations of oxygen [µM] (blue square), ammonium [µM] (dark green triangle), nitrite [µM] (pink triangle) and nitrate [µM] (light green triangle).
