# Supplementary material for: Abundance and Diversity of Denitrifying and Anammox Bacteria in Seasonally Hypoxic and Sulfidic Sediments of the Saline Lake Grevelingen
Source: Front Microbiol. 2016 Oct 20;7:1661. doi: 10.3389/fmicb.2016.01661 (PMC5071380; doi:10.3389/fmicb.2016.01661)
Supplement: Supplementary file 7 [file Image2.PDF]

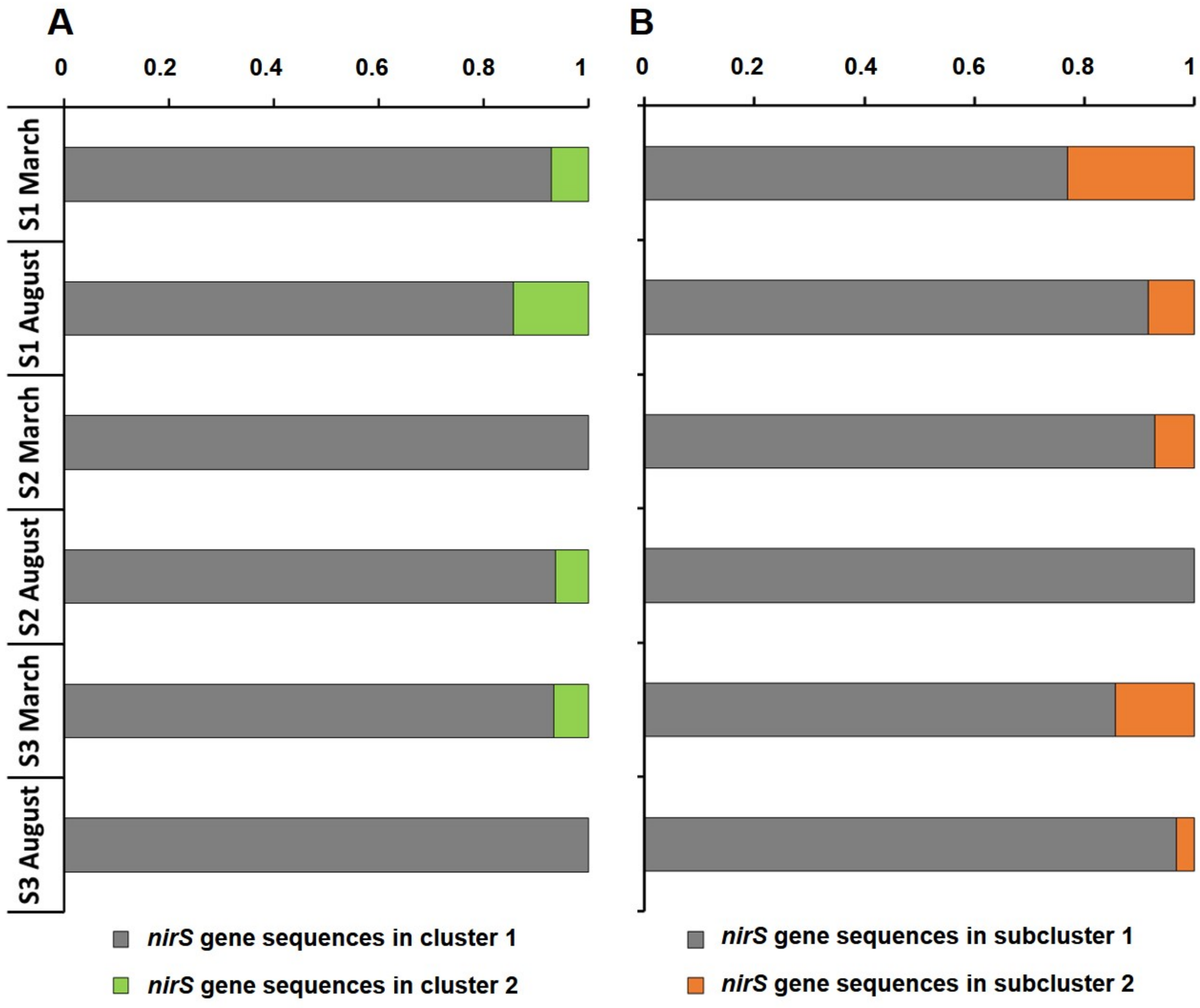

Figure S2: A) Relative percentage of *nirS* sequences of denitrifying bacteria in cluster 1 and 2 and B) in subcluster 1.1 and 1.2 according to the three stations in March and August.
