# Supplementary material for: Abundance and Diversity of Denitrifying and Anammox Bacteria in Seasonally Hypoxic and Sulfidic Sediments of the Saline Lake Grevelingen
Source: Front Microbiol. 2016 Oct 20;7:1661. doi: 10.3389/fmicb.2016.01661 (PMC5071380; doi:10.3389/fmicb.2016.01661)
Supplement: Supplementary file 8 [file Image3.PDF]

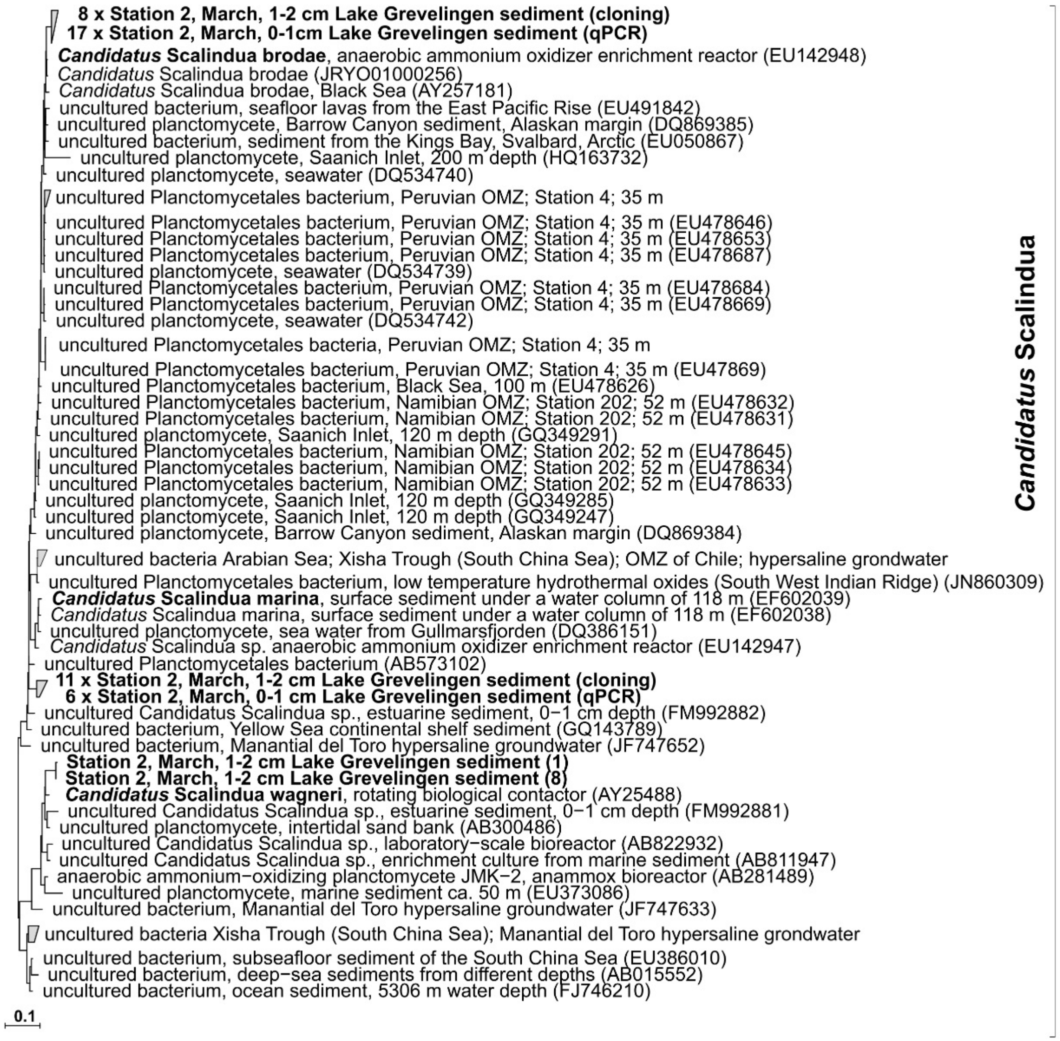

Figure S3: Phylogenetic tree of anammox bacteria partial 16S rRNA gene sequences retrieved in this study (21 DNA sequences recovered by amplification (PCR) and cloning and 21 DNA sequences recovered by amplification (qPCR) and cloning from sediments (0–1 cm) of station S2 in March) and closest relatives (bold: our sequences and closest known relatives); the scale bar indicates 10% sequence divergence.
